# Harnessing Redox Disruption to Treat Human Herpesvirus 8 (HHV-8) Related Malignancies

**DOI:** 10.3390/antiox12010084

**Published:** 2022-12-30

**Authors:** Adélie Gothland, Aude Jary, Philippe Grange, Valentin Leducq, Laurianne Beauvais-Remigereau, Nicolas Dupin, Anne-Geneviève Marcelin, Vincent Calvez

**Affiliations:** 1 INSERM, Institut Pierre Louis d’Epidémiologie et de Santé Publique (iPLESP), AP-HP, Department of Virology, Hôpital Pitié-Salpêtrière, Sorbonne Université, 75013 Paris, France; 2 Cutaneous Biology Lab, INSERM U1016, UMR8104, Institut Cochin, Université de Paris, 24 Rue du Faubourg St Jacques, 75014 Paris, France; 3 Department of Dermatology, CeGGID et CNR IST Bactériennes, Hôpital Cochin Site Port Royale, AP-HP, Groupe Hospitalier Paris Centre Cochin-Hôtel Dieu-Broca, 123 Boulevard de Port Royal, 75014 Paris, France; 4 SkinDermic, 75003 Paris, France

**Keywords:** HHV-8, Kaposi sarcoma, primary effusion lymphoma, metabolism, oxidoreduction disruption, oncogenesis

## Abstract

Reprogrammed metabolism is regarded as a hallmark of cancer and offers a selective advantage to tumor cells during carcinogenesis. The redox equilibrium is necessary for growth, spread and the antioxidant pathways are boosted following Reactive Oxygen Species (ROS) production to prevent cell damage in tumor cells. Human herpesvirus 8 (HHV-8), the etiologic agent of Kaposi sarcoma KS and primary effusion lymphoma (PEL), is an oncogenic virus that disrupts cell survival-related molecular signaling pathways leading to immune host evasion, cells growths, angiogenesis and inflammatory tumor-environment. We recently reported that primaquine diphosphate causes cell death by apoptosis in HHV-8 infected PEL cell lines in vivo and exhibits therapeutic anti-tumor activity in mice models and advanced KS. Our findings also suggest that the primaquine-induced apoptosis in PEL cells is mostly influenced by ROS production and targeting the redox balance could be a new approach to treat HHV-8 related diseases. In this review, we summarized the knowledge about the influence of ROS in cancer development; more specifically, the proof of evidence from our work and from the literature that redox pathways are important for the development of HHV-8 pathologies.

## 1. Introduction

Although the first reports of the metabolic changes that are typical of tumors date back over a century, the study of cancer metabolism has recently attracted new attention. Studies on cancer cell metabolism have increased our knowledge of the processes and practical effects of tumor-associated metabolic abnormalities at different phases of carcinogenesis with the use of novel biochemical and molecular biology methods. It has become clear that tumorigenesis-related metabolic changes affect all phases of cell-metabolite interaction, including (i) affecting metabolite influx by enhancing the capacity to obtain the necessary nutrients, (ii) determining how the nutrients are preferentially assigned to metabolic pathways that contribute to cellular tumorigenic properties, and (iii) exerting long-lasting effects on cellular fate, including altera.

In this review, we summarized the knowledge about the redox homeostasis and its implication in cells cancer. More specifically, we focused our work on the neoplasia associated with HHV-8 and the interest of redox balance as a potential therapeutic target for the management of these diseases.

## 2. Reprogrammed Metabolism and Cancer Cell Biology

By around 50 years, one of the most crucial fields of cancer biology study is cancer metabolism. The field is founded on the idea that cancer cells’ metabolic processes are different from those of normal cells, and that these differences help cancer cells develop and maintain their malignant characteristics. Reprogrammed metabolism is regarded as a hallmark of cancer since several changed metabolic characteristics are seen relatively often across many different kinds of cancer cells [1,2,3]. One of the main issues guiding research in the subject is how exactly metabolism is reprogrammed in cancer cells, what functions or malignant characteristics are permitted by these activities, and how to take advantage of metabolic modifications for therapeutic benefit.

Reprogrammed activities enhance cellular fitness to offer a selective advantage during carcinogenesis. The majority of the traditional examples of reprogramming activity either help cells survive under adverse circumstances or let cells to thrive and proliferate at abnormally high rates.

According to logic, if changed bioenergetics, increased biosynthesis, and redox imbalance are advantageous to the malignant cell, then some of these factors may be good therapeutic targets.

Numerous cases where suppression of an increased metabolic activity leads in reduced development of experimental tumors corroborate this interpretation of cancer metabolism [4,5]. In certain instances, the unique metabolic vulnerabilities of cancer cells have been translated into successful treatments for cancer in people. The enzyme asparaginase, which changes the amino acid asparagine into aspartic acid and ammonia, is a crucial part of acute lymphoblastic leukemia (ALL) therapy [6]. ALL cells need a steady supply of asparagine from the plasma because to their high rates of protein synthesis and inadequate capacity to generate asparagine de novo. By giving asparaginase throughout the body, this supply is largely removed. Ultimately, determining the stage of tumor development at which each route benefits the cancer cell will be necessary for metabolic treatment to be effective. Early in carcinogenesis, when the developing tumor starts to suffer nutritional restrictions, several activities become crucial [7]. In other circumstances, alterations to pathways could be unnecessary for primary tumors but necessary for metastasis [8,9]. It will be crucial to specify their context-specific functions in physiologically correct models of tumor start and development since new treatment targets are proposed from straightforward experimental models like cultured cells.

## 3. Human Herpesvirus 8 Neoplasia Is a Cell Proliferation-Causing Virus That Disrupts Cell Survival-Related Molecular Signaling Pathways

Several human malignancies, including Kaposi’s sarcoma (KS), multicentric Castleman’s disease (MCD), and primary effusion lymphoma (PEL), are caused by the oncogenic Kaposi’s sarcoma-associated herpesvirus (KSHV, also known as Human Herpesvirus 8, HHV-8). These HHV-8-associated disorders, which are all considered orphan diseases, pose significant clinical issues that are challenging to treat and have limited available treatment choices. Infection with the human immunodeficiency virus (HIV), as well as other illnesses and/or therapies related to immunological deficiencies, are usually linked to these disorders. Although combination antiretroviral treatment (cART) has been effective in improving HIV-infected patients’ outcomes, its advantages are relatively limited in patients with PEL and MCD, and KS continues to be the most frequent malignancy in HIV-infected people [10,11]. Combination cytotoxic chemotherapies are a widely used treatment for systemic KS and PEL [12]. However, systemic chemotherapy’s toxicity interacts with that of immune suppression or antiretroviral therapy, and there are alternative effective treatments available, which further reduces the effectiveness of the treatment [13,14]. Thus, there is a medical need for efficient and less harmful treatments that focus on the HHV-8 virus, infected cells, or important cellular pathways. The likelihood of success with antiviral anti-herpesvirus treatment is minimal given the low degree of virus lytic infection in PEL and KS patients [10]. In fact, HHV-8 has created a variety of mechanisms to create chronic latent infection, which continues to predominate in the majority of infected cells [15]. By allowing host immune evasion and encouraging tumor cell survival and proliferation through the production of a small number of oncogenic latent genes, HHV-8 uses latency to thwart the death of tumor cells that are infected with it [16,17]. It’s significant to note that diverse infected cell types, from primary latent infection of endothelium cells to long-term latency of lymphoma cells, exhibit highly comparable HHV-8 transcription patterns and viral survival mechanisms [18]. Therefore, a key area of focus for the elimination of HHV-8-infected cells and, eventually, the treatment of the usual HHV-8-associated malignancies, is the molecular signaling pathways implicated in the survival of infected cells.

In the current investigation, we discovered primaquine diphosphate (PQ) as a brand-new, incredibly promising targeted therapeutic medication that precisely causes cell death by apoptosis in HHV-8-infected PEL cell lines when used in vitro [19]. In an in vivo nonobese diabetic (NOD)/SCID PEL mice model and in patients with advanced KS, we demonstrated that PQ exhibits therapeutic anti-tumor activity [19].

## 4. Redox Unbalance in Cancer

ROS (Reactive Oxygen Species), including superoxide anion (O_2_), hydrogen peroxide (H_2_O_2_), and the hydroxyl radical (OH), are oxygen-containing chemical entities that are produced inside of cells [20]. The formation of O_2_ arises from the reduction of oxygen by one electron in the mitochondria and cytosolic NADPH (Nicotinamide adenine dinucleotide phosphate) oxidases (NOXs) [21]. The process that converts O_2_ into H_2_O_2_ is catalyzed by the superoxide dismutase 1 or 2, which can be located in the cytosol or mitochondrial matrix, respectively. H_2_O_2_ is then detoxified to water by the enzyme activity of mitochondrial and cytosolic peroxiredoxins (PRXs), which as a result suffer H_2_O_2_-mediated oxidation of their active-site cysteines [22]. Thioredoxin (TXN), thioredoxin reductase (TrxR), and the reducing equivalent NADPH are used to reduce oxidized PRXs in order to complete the catalytic cycle [23]. Through the oxidation of reduced glutathione (GSH) by H_2_O_2_, glutathione peroxidases (GPXs) can also convert H_2_O_2_ to water in the mitochondrial matrix and cytoplasm [24]. Finally, glutathione reductase (GR) and NADPH work together to transform oxidized glutathione (GSSG) back into GSH. Otherwise, the strong antioxidant catalase, which is present in the peroxisomes, has also the potential to detoxify H_2_O_2_ to water on its own.

However, when combined with ferrous and cuprous ions, H_2_O_2_ may quickly be transformed into OH and cause the oxidation of lipids, proteins, and DNA resulting in cellular damage. The rising of H_2_O_2_ levels could also activate the cell death signaling pathways. For the maintenance of several antioxidant defense mechanisms, NADPH is necessary. In addition to the one-carbon metabolism, the cytosol also produces NADPH from the oxidative PPP, malic enzyme 1, and IDH1. One-carbon metabolism and IDH2 have a role, in part, in the regulation of NADPH production in the mitochondria.

In the past, ROS were thought to be harmful metabolic by-products of protein folding and cellular respiration. However, research over the last two decades has uncovered a hitherto unrecognized role for ROS in cellular signaling. Low concentrations of ROS, in particular H_2_O_2_, can reversibly oxidize protein cysteine residues to stimulate cell growth and adaptability to metabolic stress [25]. Compared to normal cells, cancer cells produce more spatially confined mitochondria- and NOX-dependent ROS [26,27]. As a result, the signaling pathways PI3K and mitogen-activated protein kinase/extracellular signal-regulated kinase (MAPK/ERK), as well as the transcription factors HIF and NFkB might be activated proximally, both being required for the carcinogenic process. Oncogenic lesions and the tumor microenvironment both contribute to the increased rate of spatially localized ROS generation that is particular to cancer cells. For instance, in cancer cells, hypoxia (low oxygen levels) and the activation of oncogenes induce the increased rate of ROS generation from the mitochondria and NOXs in cancer cells [28,29,30]. Antioxidants that target the mitochondria and NOX inhibitors can stop tumor growth, hypoxic activation of HIF, metastasis, and cancer cell proliferation [31,32,33].

The enhanced production of antioxidant proteins is required to prevent the elevated localized ROS from reaching levels that cause cellular damage in cancer cells. This increased localized ROS in cancer cells stimulates signaling pathways and transcription factors to support carcinogenesis [34]. In that perspective, cancer cells also have higher levels of ROS scavenging enzymes than normal cells do, in order to prevent ROS-mediated activation of death-inducing pathways such as c-Jun N-terminal kinase (JNK) and p38 MAPK and oxidation of lipids, proteins, and DNA, which causes permanent damage and cell death. By activating the transcription factor nuclear factor (erythroid-derived 2)-related factor-2 (NRF2), cancer cells might boost their antioxidant capacity [35]. NRF2 is specifically activated once the contact between NRF2 and its binding partner Kelch-like ECH-associated protein 1 is broken (KEAP1). Critical KEAP1 cysteine residues are susceptible to oxidation, succination, and glutathionylation, which prevents the KEAP1-NRF2 connection and causes NRF2 to be degraded by proteasomes. NRF2 activation is also possible without KEAP1 participation [36]. GPXs and TXNs, along with enzymes involved in GSH production and cysteine import via the cystine/glutamate antiporter, are only a few of the antioxidant proteins that NRF2 stimulates the transcription of after it has been active. To keep GPXs and TXNs’ antioxidant abilities, NADPH is also required and NRF2 is crucial for triggering the enzymes that raise cytosolic NADPH levels. Additionally, the serine biosynthesis pathway, which generates NADPH in the mitochondria and is crucial for preserving redox balance in hypoxic environments, is also controlled by NRF2 [37,38]. Therefore, blocking NRF2 or antioxidative proteins would allow ROS to accumulate to dangerous levels and prevent the proliferation of cancer cells [36,39,40].

**Figure 1:**
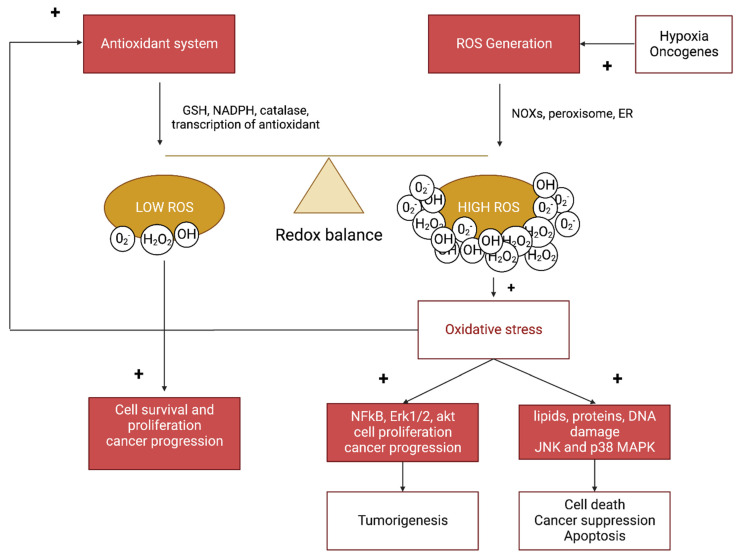
Schematic representation of the redox homeostasis in cancer cells. The redox balance is crucial for normal and cancer cells survival. In normal condition, an increase of ROS generation leads to the activation of different pathways involved in programmed cell death. In cancer cells, metabolism is disrupted and lead to an increase of ROS levels which can also activate different pro oncogenic pathways inducing cell survival and proliferation. To thwart the high rates of ROS, the oxidative stress can also stimulates the antioxidant system to reduce ROS levels and prevent the cells from cell death.

A schematic representation of the redox balance in cancer cells is presented in Figure 1.

Redox equilibrium is necessary for the growth and spread of tumors. A growing idea of redox balance postulates that the metabolic activity of cancer cells rises as a tumor grows [41]. Because of this, ROS production increases, which activates signaling pathways that support cancer cells survival, proliferation and metabolic adaptation [34]. As a result, tumor cells boost their antioxidant ability to prevent harmful levels of ROS, enabling the growth of cancer [42]. Low glucose levels restrict the amount of oxygen that can pass through the cytosolic oxidative PPP, increasing ROS levels brought on by the hostile tumor microenvironment. The result is a decrease in cytosolic NADPH levels. In these nutrient-deficient situations, cells activate AMPK to raise NADPH levels by promoting PPP-dependent NADPH and decreasing anabolic processes, such as lipid synthesis, that need high NADPH concentrations [43,44]. For a tumor to spread, increased mitochondrial respiration and ROS-dependent signaling are also necessary [32,45]. However, when tumor cells detach from a matrix and are exposed to high levels of ROS that result in cellular damage, they must activate adaptative ROS-mitigating pathways in order to survive and grow [46,47]. Distant metastasis is enabled by the capacity to up-regulate antioxidant proteins and boost flow via NADPH-producing metabolic pathways. According to these findings, it would be advantageous to disable the antioxidant ability of cancer cells to increase ROS levels in order to avoid spreading.

## 5. ROS and Kaposi Sarcoma

Risks factors associated with the transmission of HHV-8 and the development of related diseases remain debated in the literature.

In epidemiological studies, poppers have been described as a risk factor for the transmission of HHV-8 in men having sex with men population [48,49], but also in the development of KS, even if no dose-dependent association has been found [50]. In vitro, we also reported that poppers could induce HHV-8 replication after a short time of incubation on PEL BC-3 cell line [51]. Physiologically, amyl nitril could either behave like ROS due to the presence of oxygen in its molecular structure [52,53], or induce their production, as it was reported for certain drugs used in therapeutics, i.e., bortezomib and placlitaxel [54,55], in order to induce HHV-8 viral replication. Furthermore, amyl nitrile was reported to induce methemoglobinemia in patients using poppers as recreational drugs. It was hypothesized that it occurred directly through their activity as oxidizing agents [52]. The increase of methemoglobinemia might also induced locally hypoxia and thus indirectly, promotes HHV-8 reactivation [56].

In the environment, several chemical compounds have also been described as risk factor for the development of classic or endemic Kaposi sarcoma [57]. Patients residing in regions with soils rich in iron, aluminum and silica, particularly near volcanoes, have an increased risk of developing KS [58,59]. Furthermore, studies have shown that on the one hand, iron can function as a catalyst for the generation of ROS under pathological conditions and on the other hand that the use of an iron chelator (i.e., deferoxamine) controls the development of Kaposi sarcoma in vitro [60]. However in mice and human studies, the use of iron chelator induce the opposite effect with a growth of the Kaposi sarcoma lesion, suggesting that this molecule could also induce an anti-apoptotic mechanism to stimulate lesion growth [61,62].

Thus, there is epidemiological evidence that compounds with chemical structure near ROS or involving redox may contribute to the development of Kaposi sarcoma.

## 6. Modulating ROS Balance to Fight against HHV-8 Associated Diseases

The need for a more specialized therapeutic strategy still exists because the efficacy of the available treatments for HHV-8-related disorders is limited [63]. The antimalarial primaquine diphosphate was originally found in our laboratory following a thorough pharmacological screening as a possible targeted treatment agent to treat HHV-8-associated PEL and KS. As opposed to HHV-8-uninfected cells, we were able to establish in vitro that primaquine is selective to HHV-8-infected PEL cell lines and generates cytotoxic effects through executioner caspases-3/-7-dependent apoptosis. Understanding how primaquine causes apoptosis, we showed that caspase-8 and -9 are activated, demonstrating the participation of both intrinsic and extrinsic apoptosis pathways in primaquine-induced cell death in PEL cells.

As apoptotic triggers and modulators of cell death, ROS and oxidative stress are well-known [64,65].

**Figure 2:**
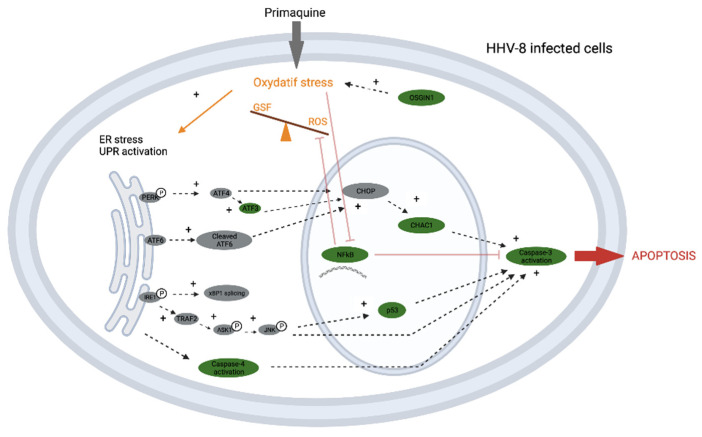
Schematic representation of primaquine action on KSHV-infected cells to induce apoptosis. Oxidative stress induced by the primaquine leads to an increase of ROS and decrease of the GSF. In response to the oxidative stress, the pro-apoptotic UPR pathway is activated and induced the up-regulation of the ATF3, CHAC-1 and OSGIN1 transcripts. Caspase-4 pathway could also be activated directly through the activation of the UPR pathway. The oxidative stress induced by primaquine could also inhibated the NFkB pathway, leading to the lifting of the negative retrocontrole on the expression of the caspase-3. Altogether, our result suggest that the primaquine could induced apoptosis on KSHV-infected cells by stimulating different pro-apoptotic pathways.

We assessed primaquine’s pro-oxidant action in PEL cells since primaquine-induced oxidative stress has been shown to be strongly connected to the hemolytic toxicity of primaquine and certain of its metabolites in G6PD-deficient erythrocytes [66,67]. We demonstrated that GSH depletion and intracellular ROS production were both enhanced by primaquine. N-acetylcysteine, an antioxidant, was used as a pre-treatment to significantly reduce ROS production and to mitigate the cytotoxicity and caspases-3/-7 activation caused by primaquine. These findings suggested that the primaquine-induced apoptosis in PEL cells is mostly influenced by ROS production. This result was confirmed by the up regulation of OSGIN1 expression seen in PEL cells treated with primaquine. OSGIN1 is a tumor suppressor gene that is activated by oxidative stress and has been previously reported to play a role in the apoptosis of PEL cells, in part through the control of ROS production and GSH synthesis [68] (Figure 2).

According to earlier research, large amounts of ROS mostly caused cell death, which is why they are crucial for controlling the balance between HHV-8 reactivation and PEL cell death [53]. We noticed that extracellular HHV-8 virion generation was boosted after a prolonged 48-h incubation of BC-3 cells with primaquine. Primaquine caused a significant amount of cell death within 48 h, thus it seems likely that the virus would detect these signals and use them to flee from the dying cells. It is unclear, nevertheless, whether these virions originated from enhanced release of HHV-8 virions in lytic PEL cells or from the viral reactivation of latent cells. It is significant that our findings did not reveal any direct effects of primaquine until 24 h after treatment, even when apoptotic pathways that cause PEL cell death had already been active. Furthermore, we found no evidence of an increase in HHV-8 viral load in either the ascites or the intraperitoneal solid sample from primaquine-treated NOD/SCID PEL xenograft mice. These results clearly suggest that viral lytic replication was not necessary for primaquine’s induction of cell death.

RNA-seq investigation brought to light the activation of some hallmarks of the endoplasmic reticulum (ER) stress-mediated apoptosis signaling pathway as we studied the cell death signaling pathways associated with primaquine modes of action. The increased protein requirement of cancer cells can cause ER stress and, as a result, the unfolded protein response (UPR) to maintain ER homeostasis, which promotes tumor cell survival due to their accelerated rate of growth and proliferation [69,70]. However, the pro-apoptotic pathways of the UPR are activated, including the production of the pro-apoptotic transcription factor CHOP and activation of caspase-4, which results in apoptosis if the ER stress is too severe, persistent, or cannot be addressed [71,72,73,74,75]. As a result, ER stress-targeting treatment provides an intriguing approach for anti-tumor, including anti-PEL, therapies by inducing pro-apoptotic pathways of the UPR [69,76,77,78,79]. Our research revealed that primaquine-treated PEL cells also had elevated expression of ATF3 and CHAC1, as well as a targeted induction of CHOP, in addition to ER stress-dependent caspase-4 activation. Downstream of ATF3 and CHOP, CHAC1 is a pro-apoptotic ER stress-inducible gene that can partially mediate the pro-apoptotic effects of both of these transcription factors [80,81,82]. The molecular basis of the activation of the ER stress-mediated apoptotic signaling will require more research but our findings might suggest that primaquine-induced apoptosis in HHV-8-infected cells is produced by activation of the pro-apoptotic ER stress-inducible gene.

It has been revealed that HHV-8 expression patterns and viral latency processes are remarkably identical in every infected cell [18,83], which contributes to HHV-8-induced pathogenesis and malignancies. Therefore, we speculate that a compound that targets signaling pathways linked to latently infected cells, cell death will eliminate various latently infected cell types, resulting in the treatment of HHV-8-related cancers. An exploratory proof of concept clinical trial on Kaposi’s sarcoma was carried out with the help of our in vitro and in vivo primaquine anti-tumor efficacy and safety findings in PEL cells and mouse models, as well as the fact that primaquine has been used all over the world since the 1950s with remarkable tolerance among glucose 6-phosphate dehydrogenase (G6PD)-normal patients [84]. Primaquine was well tolerated and showed some promising anti-tumor activity in this pilot clinical investigation on Kaposi’s sarcoma-related lesions and lymphoedema. These findings support expanding the clinical study to include more patients with KS and/or PEL receiving a variety of primaquine doses to more thoroughly assess the drug’s therapeutic effectiveness. Importantly, giving primaquine medication prior to a thorough G6PD deficiency diagnosis would significantly lower the risk of drug-induced acute hemolytic anemia in susceptible people.

## 7. Rational for Combining Different Therapeutic Approach in HHV-8 Associated Diseases Treatment

As it is now well known, tumors grow and evolve trough a constant crosstalk with the surrounding microenvironment [85], and emerging evidence indicates that angiogenesis and immunosuppression frequently occur simultaneously in response to this crosstalk. For instance, hypoxia in cancer cells can promote ROS generation, assumed leading to programmed death cell [86]. On the other hand, hypoxia also stimulates angiogenic factors to ensure sufficient nutrient and oxygen supply and allow metastatic spread. [87]. Several anti-angiogenic drugs, such as anti-vascular endothelial growth factor (VEGF) has been approved alone or in combination for cancer treatment. Indeed, angiogenesis is now considered a driver of immunosuppression and immune evasion in the tumor microenvironment [88]. However, fast enough, resistance to anti-angiogenic therapies has been reported suggesting that the association of multiple anti-angiogenic molecules or a combination of anti-angiogenic drugs with other treatment regimens would be benefit to overcome this resistance. For instance, Ribatti et al. reported that combining anti-VEGF and immunotherapy might have the potential to tip the balance of the tumor environment and improve treatment response [87].

In Kaposi sarcoma, lesions are characterized by a proliferation of spindle cells infected with HHV-8, neo angiogenesis, inflammation and immune cells infiltration. On its own, the HHV-8 genome code for a plethora of viral factors, including proteins and non-coding RNAs, some of which have been shown to deregulate angiogenic pathways and promote tumor growth [89]. Several anti-angiogenic drugs has been tested to treat Kaposi sarcoma, and the most interesting results were obtained with the thalidomide and its deritatives and the inhibitors of the mTOR pathway as sirolimus (rapamycin) and temsirolimus [63]. Still several cases relapse, for instance epidemic Kaposi sarcoma in patients under efficiency cART [90]. In regards to the results described in several cancers and considering that HHV-8 associated diseases make no exception in the constant crosstalk with the surrounding environment, we assume that combining the primaquine with other therapies might be more efficient in patients with severe and recurrent Kaposi sarcoma.

## 8. Conclusions

In order to support the survival, development, and advancement of cancer, ROS formation, signaling, and control are changed physiological processes. A deeper comprehension of the function of redox balance in cancer biology is anticipated to offer novel targets for the prevention of cancer, the avoidance of treatment resistance, and the achievement of clinically advantageous outcomes. According to the nature and stage of the tumor, the reliance on antioxidant pathways can change. Inter- and intra-tumoral genetic and metabolic heterogeneity in malignancies is a crucial factor to take into account. Targeting redox dependency in one subpopulation of cancer cells may spare other subpopulations Additionally, there are differences in the redox regulation of tumor-resident cells and cells in circulation that are headed for colonization by metastatic cells. Consequently, a comprehensive understanding of the ROS axis will aid in identifying the potential and difficulties in creating ROS-based therapies that are more clinically successful.

These approaches can be used in the treatment of HHV-8 related diseases with for example of the antimalarial primaquine diphosphate that causes cell death by inducing apoptosis, specifically in infected with HHV-8. This is done by activating apoptosis pathways that are mediated by oxidative stress and ER stress. This compound has to be developed, using other galenic formulations, as a novel, promising targeted therapeutic agent in the treatment of, at least, HHV-8-associated PEL and KS because of its dose-dependent, anti-tumor efficacy in vivo in a PEL mouse model and in patients with severe KS as well as its good tolerance and lack of significant side effects. For severe and recurrent forms of KS, combining primaquine with others therapies could be relevant.

## Data Availability

Not applicable.
